# A gene expression profile indicative of early stage HER2 targeted therapy response

**DOI:** 10.1186/1476-4598-12-69

**Published:** 2013-07-01

**Authors:** Fiona O’Neill, Stephen F Madden, Martin Clynes, John Crown, Padraig Doolan, Sinéad T Aherne, Robert O’Connor

**Affiliations:** 1 Molecular Therapeutics for Cancer Ireland, National Institute for Cellular Biotechnology, Dublin City University, Glasnevin, Dublin 9, Ireland; 2 School of Nursing and Human Sciences, Dublin City University, Glasnevin, Dublin 9, Ireland

**Keywords:** Breast cancer, HER2 predictive markers, Targeted therapies

## Abstract

**Background:**

Efficacious application of HER2-targetting agents requires the identification of novel predictive biomarkers. Lapatinib, afatinib and neratinib are tyrosine kinase inhibitors (TKIs) of HER2 and EGFR growth factor receptors. A panel of breast cancer cell lines was treated with these agents, trastuzumab, gefitinib and cytotoxic therapies and the expression pattern of a specific panel of genes using RT-PCR was investigated as a potential marker of early drug response to HER2-targeting therapies.

**Results:**

Treatment of HER2 TKI-sensitive SKBR3 and BT474 cell lines with lapatinib, afatinib and neratinib induced an increase in the expression of *RB1CC1*, *ERBB3*, *FOXO3a* and *NR3C1*. The response directly correlated with the degree of sensitivity. This expression pattern switched from up-regulated to down-regulated in the HER2 expressing, HER2-TKI insensitive cell line MDAMB453. Expression of the *CCND1* gene demonstrated an inversely proportional response to drug exposure. A similar expression pattern was observed following the treatment with both neratinib and afatinib. These patterns were retained following exposure to traztuzumab and lapatinib plus capecitabine. In contrast, gefitinib, dasatinib and epirubicin treatment resulted in a completely different expression pattern change.

**Conclusions:**

In these HER2-expressing cell line models, lapatinib, neratinib, afatinib and trastuzumab treatment generated a characteristic and specific gene expression response, proportionate to the sensitivity of the cell lines to the HER2 inhibitor.

Characterisation of the induced changes in expression levels of these genes may therefore give a valuable, very early predictor of the likely extent and specificity of tumour HER2 inhibitor response in patients, potentially guiding more specific use of these agents.

## Introduction

Overexpression of the epidermal growth factor (EGFR) family of proteins has been demonstrated to have significant negative therapeutic significance for breast cancer. This group of proteins is comprised of EGFR, HER2, HER3 and HER4 [1]. In the development of targeted therapies, the efficacy of EGFR and HER2 inhibitors has been demonstrated. Erlotinib and gefitinib have been the two most successfully developed and widely-used targeted EGFR-targeting drugs. Both gefitinib and erlotinib have been used in the treatment of cancers that harbour an EGFR mutation [1], particularly non-small cell lung cancer (NSCLC).

HER2-positive breast cancer, in which the HER2 receptor is either overexpressed and/or amplified, account for approximately 20-30% of human breast cancers [2] and are associated with poorer prognosis [3,4]. Non-targeted breast cancer treatment options may include one or more of chemotherapy, radiation, and surgery, while HER2 overexpressing breast cancers will typically involve trastuzumab-based therapy with newer agents such as lapatinib, providing a second line for treatment [2,5].

Lapatinib was one of the first HER2-targetting tyrosine kinase inhibitors (TKI) to be used in the clinic [6]. This dual-kinase inhibitor which also targets EGFR was developed by GlaxoSmithKline (GSK) and is currently FDA approved for the treatment of refractory breast cancer in combination with capecitabine [7]. Identification of robust, reproducible predictive biomarkers is vital for the appropriate application of such therapies. A number of recent publications have found a correlation between pTEN/AKT/PI3K pathway activation (as assessed using protein-based technology) and the response the patient to either traztuzumab or lapatinib. The consensus of these reports is that patients demonstrating low pTEN expression are likely to exhibit resistance to traztuzumab but sensitivity to lapatinib. A role for receptor autophosphorylation and down-stream regulators of apoptosis has also been shown to be important [8-10]. These studies have provided a valuable insight into intrinsic resistance in the HER2 target models but have limited application as more broadly clinically useful predictive biomarkers of response to therapy.

More recently the small molecule TKI therapeutic arsenal has seen the addition of newer agents such as, afatinib and neratinib. Afatinib is an irreversible EGFR/HER2 inhibitor developed by Boehringer Ingelheim [11] currently being clinically evaluated in NSCLC. The aniline-quinazoline structure of the inhibitor has the potential to irreversibly bind to the EGFR and HER2 receptors, which in turn prevents activation of the kinase domain [11-13].

Similar to afatinib, neratinib is also an irreversible inhibitor of the EGFR and HER2 receptors. Developed by Wyeth, this small molecule also inhibits the HER4 receptor [14]. Neratinib interferes with phosphorylation by binding to the cytoplasmic domain of the receptors resulting in the inhibition of downstream phosphorylation of substrates. This inhibition in turn has an effect on the cells ability to proliferate and can ensure that the cell arrests at the correct cell cycle transition to ensure cell death occurs [15,16].

Due to their ability to potently inhibit EGFR, both afatinib and neratinib have been assessed in lung cancer that has become resistant to gefitinib and erlotinib due to the T790M point mutation in the kinase domain [11,14].

In a previous publication by our group, we identified a panel of genes whose expression in response to 12 hours of lapatinib treatment altered in a manner proportionate to the sensitivity of the cell-lines assessed to this agent [17]. Co-inertia analysis was used to evaluate microarray data from untreated and lapatinib treated BT474 and SKBR3. A panel of 27 genes were validated using RT-PCR and from this analysis, genes that had a differential expression of ±2 were considered significant. This multivariate statistical technique is used to link transcription factor binding site (TFBS) target predictions and gene expression data in order to identify transcription factors (TF) associated with the cellular response to lapatinib [18,19]. CIA allowed us to identify commonality between the expression of the genes and the TFs that are predicted to target these genes. Using this gene panel of five (*RB1CC1*, *FOXO3a*, *NR3C1*, *ERBB3* and *CCND1*), we examined the differential expression of these genes in response to pharmacologically relevant concentrations of neratinib, afatinib and traztuzumab to characterise if this panel informed on the sensitivity of the cell models to lapatinib alone or might also be useful in predicting cellular response to other HER2-targetting therapies. Better prediction of the likely efficacy of a targeted therapy could have huge implications for improved efficacy of cancer treatment, patient-individualised optimisation of the available arsenal of treatment options and, through rapid identification of likely response/non-response, greatly reducing the overall financial burden of these expensive but sometimes lifesaving pharmaceuticals.

## Materials and methods

### Drug preparations

Lapatinib tosylate, neratinib, afatinib, dasatinib and gefitinib were all sourced from Sequoia Chemicals Inc. The drugs were prepared to 10 mM in DMSO. Traztuzumab was sourced from Roche, Basel, Switzerland and epirubicin was sourced from Pfizer, New York, NY, USA. 5dFUR, an active metabolite derivative of capecitabine, was sourced from Sigma, St Louis, MO, USA. As with the TKI drugs, the 5dFUR was prepared in DMSO.

### Cell culture

The cell lines that were examined were BT474 and SKBR3, HER2-overexpressing, lapatinib-sensitive breast cancer cell lines, and MDAMB453, a HER2-overexpressing but lapatinib-insensitive breast cancer cell line. SKBR3 and MDAMB453 breast cancer cell lines were maintained in RPMI 1640 medium supplemented with 10% fetal bovine serum (PAA Labs, Austria). BT474 cells were maintained in Dulbeccos Modified Eagles medium (DMEM) supplemented with 10% fetal bovine serum, 2% L-glutamine (Sigma, St Louis, MO, USA) and 1% Sodium Pyruvate (Sigma). All cell lines were kept at 37°C in 5% CO_2_/95% air humidified incubators.

### Drug treatment and RNA extraction

Triplicate samples were grown to approximately 75% confluency. Treated samples were conditioned with 1 μM lapatinib, 150 nM afatinib and 150 nM neratinib for 12 hours and 36 hours. Cell lines were also treated with 1 μM gefitinib for 12 hours. Control samples remained untreated for the same time period. After the cells were conditioned, the control and treated samples underwent RNA isolation using a Qiagen RNeasy mini Kit (Qiagen, Hilden, Germany) according to the manufacturer’s protocol and treated with Qiagen RNase-free DNase. cDNA template was then prepared from 2 μg of total RNA using an Applied Biosystems high capacity RNA to cDNA kit (Applied Biosystems, Foster City, CA, USA).

### Taqman RT-PCR

TaqMan gene expression experiments were performed in 10 μl reactions in Taqman Array 96 well fast plates which had been pre-seeded with assays for the genes of interest. 40 ng of cDNA template and 5 μl of Taqman fast Universal Master Mix (2×), no AmpErase UNG (Applied Biosystems, Foster City, CA, USA) were dispensed into each well. The following thermal cycling specifications were performed on the 7900HT Fast Real-Time PCR system (Applied Biosystems, Foster City, CA, USA); 20 s at 95°C and 40 cycles of 3 s at 95°C and 30 s at 60°C. Expression values were calculated using the comparative cycle threshold (C_t_) method [20]. Glyceraldehyde-3-phosphate dehydrogenase (GAPDH) was selected as the endogenous control.

### *In vitro* proliferation assay

Cells were cultured in 96 well flat bottomed plates for 24 h before they were exposed to a range of concentrations of the targeted therapies for 6 days. The % cell survival was then determined using an Acid Phosphatase assay [21]. Briefly, media was removed from plates, the wells were washed twice with PBS and the cells were exposed to 10 mM PNP substrate in 0.1M sodium acetate for approximately 1 hour. The reaction was stopped using 1M NaOH and the plates were read at 405 nm and 620 nm on the plate reader (Synergy HT, Bio-Tek, Winooski, VT, USA). The % cell survival was calculated as a percentage of non-treated controls.

### Statistical analysis

Differences in the gene expression level between untreated and drug treated samples were assessed using the Students *t* test.

## Results

### Toxicological analysis of lapatinib, afatinib and neratinib in the cell line panel

IC_50_ values were determined for lapatinib and were found to correlate with previously described values [2,17] for the 3 cell lines (BT474, SKBR3 and MDAMB453). The results are summarised in Table 1.

**Table 1:** IC50 values of selected cell lines for the panel of TKI

	**Cell line name**	**IC**_**50 **_**± SD (μM) lapatinib**	**IC**_**50 **_**± SD (μM) neratinib**	**IC**_**50 **_**± SD (μM) afatinib**
**Lapatinib Sensitive Cell Lines**	BT474	0.036 ± 0.015	0.0019 ± 0.00046	0.00323 ± 0.00075
	SKBR3	0.080 ± 0.017	0.00226 ± 0.00008	0.0075 ± 0.005
**Lapatinib Insensitive Cell Line**	MDAMB453	6.08 ± 0.825	0.820 ± 0.140	1.59 ± 0.179

### Five genes are consistently dysregulated following treatment with HER2 targeted therapies

Using Taqman PCR, the expression of five genes previously described [17] as being consistently proportionality altered in response to 12 hrs of 1 μM lapatinib treatment (*RB1CC1*, *FOX3A*, *NR3C1*, *ERBB3* and *CCND1*), were examined in response to 150 nM concentrations of afatinib and neratinib for the same time period using untreated cells as controls. BT474 and SKBR3 had the highest level of differential expression of the genes in the previous study [17] while MDAMB453 cells showed a markedly different pattern in the differential expression of the these genes.

**Figure 1:**
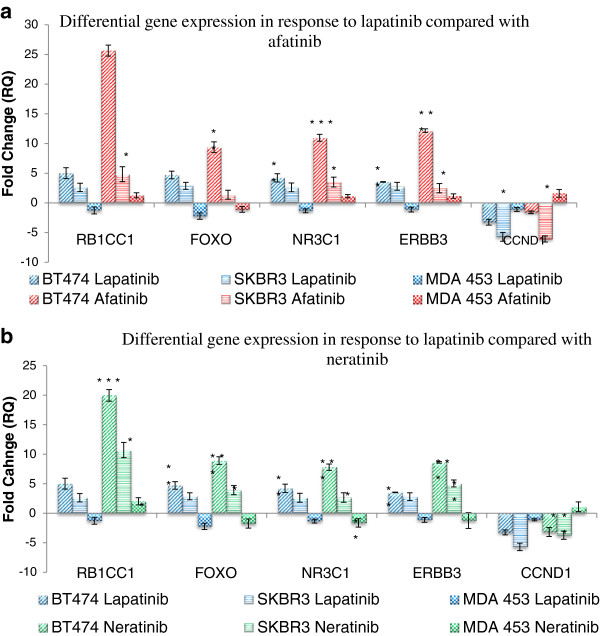
**Differential gene expression of the five genes in response to 1 μM lapatinib and a) 150 nM afatinib b) 150 nM neratinib treatment.** Assessing the 3 cell lines shows that the response to afatinib is similar to the response profile of lapatinib. N=3 **b)** Differential expression of the five genes in response to 1 μM lapatinib and 150 nM neratinib. Analysis across the 3 cell lines shows that the response to neratinib is similar to the response profile of lapatinib. N=3 * indicates p < 0.05, ** indicates p < 0.01, *** indicates p < 0.005.

Following treatment with afatinib or neratinib, the gene expression profile of *RB1CC1*, *FOX3A*, *NR3C1*, *ERBB3* and *CCND1* followed the same trends as that seen in response to lapatinib. In BT474 and SKBR3 cell lines, there was an up-regulation in the expression of *RB1CC1*, *FOX3A*, *NR3C1* and *ERBB3* and a down-regulation in the expression of *CCND1*. In MDAMB453 the expression of the five genes was shown to be either down-regulated or unchanged following the treatment with afatinib. It should be noted that in the case of the BT474 cell line, the magnitude of the differential expression was somewhat greater in the afatinib-treated cell than the lapatinib-treated cells (Figure 1).

**Figure 2:**
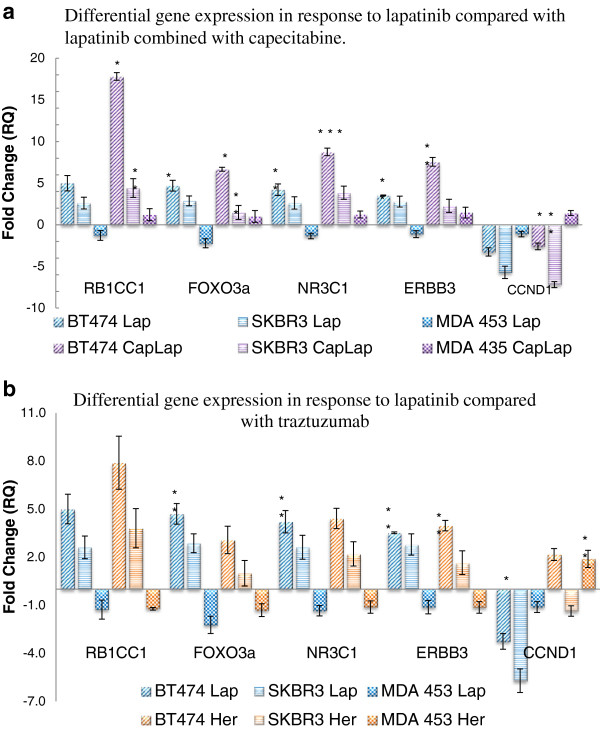
**Differential gene expression of the five genes following 1 μM lapatinib and 1 μM lapatinib in combination with 20 μM capecitabine. a)** Analysis indicates that the addition of the 5DFUR does not mask the trend evident in the lapatinib only treated cell lines N=3 **b)** Differential gene expression comparison of the five genes following 1 μM lapatinib and 150 nM traztuzumab. Analysis across the 3 cell lines indicates that there is a similar expression pattern following treatment with traztuzumab. N=3 * indicates p < 0.05, ** indicates p < 0.01, *** indicates p < 0.005.

Figure 2 shows the expression of the genes of interest in the panel of cell lines following 12 hour treatment with other approved treatments for HER2 positive breast cancer, in particular trastuzumab and lapatinib in combination with capecitabine. For the purpose of this study, 5dFUR, the active metabolite derivative of capecitabine, was used. The gene expression pattern observed in response to the FDA approved treatment regimens showed a similar trend to that seen in response to the HER2 targeting TKIs.

### Treatment of cells with non-HER2 targeted TKIs or chemotherapy reagents produces a different gene expression response

**Figure 3:**
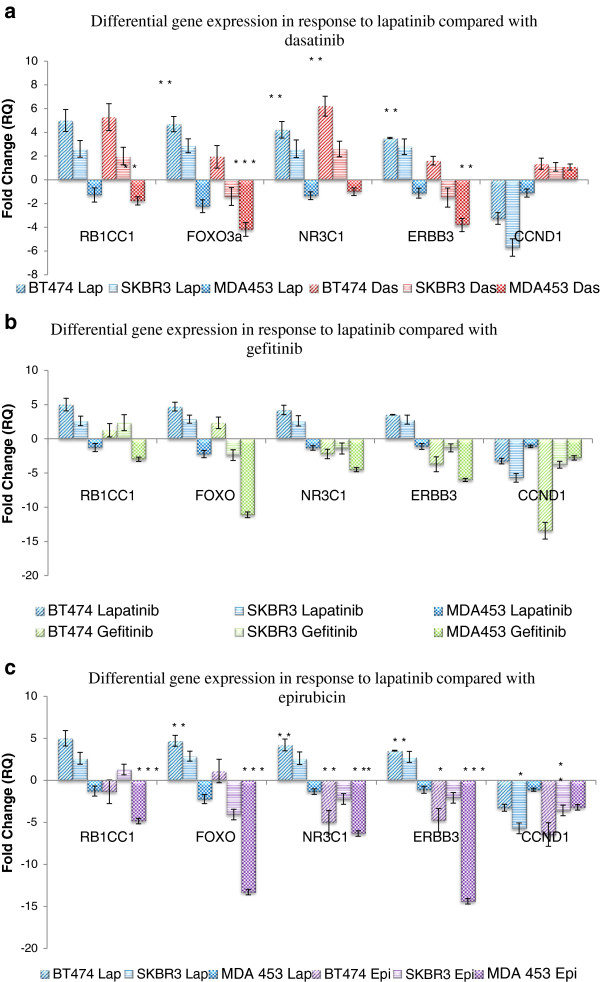
**Differential gene expression of the five significant genes following 1 μM lapatinib treatment and a) 1 μM dasatinib, b) 1 μM gefitinib and c) 25 nM epirubicin treatment.** Analysis across the 3 cell lines shows that the response to gefitinib, dasatinib and epirubicin, unlike afatinib and neratinib, does not follow the same differential gene expression profile that was indicated in response to lapatinib. N=3 * indicates p < 0.05, ** indicates p < 0.01, *** indicates p < 0.005.

In order to examine if the gene expression profile exhibited by the cell lines following lapatinib, afatinib and neratinib treatment was specifically the result of the HER2 pathway being inhibited, cells were treated with non-HER2 targeting agents 1 μM gefitinib, 1 μM dasatinib and 25 nM epirubicin for 12 hours. Gefitinib is an EGFR inhibitor that is used in the treatment of NSCLC. Dasatinib is a BCR/ABL and src family tyrosine kinase inhibitor used in the treatment of chronic myeloid leukaemia and acute lymphoblastic leukaemia [22]. Epirubicin is an anthracycline chemotherapeutic agent used in the treatment of a number of malignancies including breast and ovarian cancer. When the gene expression profile of the gefitinib, dasatinib and epirubicin-treated cells was compared to that of the lapatinib-treated cells, there was no continuation of the trends that were seen with the lapatinib treatment (Figure 3a-c).

### Gene expression changes remain consistent up to 36 hrs post treatment with lapatinib, afatinib, and neratinib

To determine if the gene expression changes shown in response to the panel of TKIs were stable over a longer time period, cells were treated for 36 hours with the same concentrations. Using RT PCR, thel mRNA levels of the target genes were further evaluated and compared to the 12 hour post treatment profiles.

**Figure 4:**
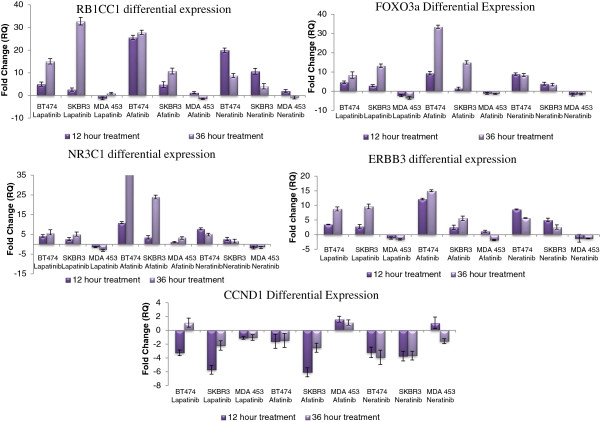
**Differential expression of the five genes in response to 1 μM lapatinib, 150 nM afatinib and 150 nM neratinib following both 12 and 36 hour exposure to the drugs.** N=3.

For *RB1CC1*, *FOXO3a*, *NR3C1* and *ERBB3* in the lapatinib- and afatinib-treated cells there was an increase in the magnitude of up-regulation in the BT474 and SKBR3 cell lines, while in the MDAMB453 cell line the expression of the genes remained unchanged or slightly more down-regulated in response to the treatment (Figure 4). In the neratinib-treated cell lines, the same trend was evident in the BT474 and SKBR3 cell results with a large increase in gene expression albeit the extent of this increase varied somewhat over the time course of the experiment. As with the other treatments, in the MDAMB453 cells the gene expression levels remained unchanged or down-regulated 36 hour post treatment.

Expression of the *CCND1* gene in the lapatinib-treated BT474 and the SKBR3 cell lines continued to be down-regulated 36 hour post treatment. In the MDAMB453 cells the gene expression remained unchanged in response to the 36 hour drug treatment. For the afatinib and neratinib-treated BT474 and SKBR3 cell lines the gene expression changes remained down regulated 36 hour post treatment of the drugs. As was the case with the other four genes, the expression pattern remained largely unchanged between treated and untreated cells (either drug) in the MDAMB453 cells.

## Discussion

In this paper, we aim to further examine the significance of our prior finding of a characteristic five gene expression response to lapatinib treatment. To do this we characterised the impact of two other HER2-targetting TKIs; afatinib and neratinib on these genes changes, and the durability of this response over different time points. In addition, we assessed the gene changes in response to two further approved treatments for HER2-positive breast cancer; trastuzumab, and lapatinib in combination with capecitabine. Finally, to evaluate how HER2-centric the changes were, we interrogated gene expression changes in response to the EGFR inhibitor, gefitinib, the BCR/ABL and Src inhibitor, dasatinib, and the anthracycline agent epirubicin [17]. BT474, SKBR3 and MDAMB453 cell lines were treated with 150 nM afatinib and neratinib for 12 hours and the gene expression analysed using RT-PCR. In line with the previously reported lapatinib treatment finding, in our panel of five genes, four *RB1CC1*, *NR3C1*, *FOXO3A* and *ERBB3* were also up-regulated in response to other HER2 inhibitor treatment;. The magnitude of the expression of these genes was correlated with the sensitivity of cells to the drug. *CCND1* was shown to be down-regulated in response to the drug treatment, again consistent with the previously published lapatinib data. Briefly, the known functions of the genes vary from altering cell cycle progression (*RB1CC1* and *CCND1*) to modulation of other transcription factors (*NR3C1*) to involvement in tumourgenesis (*FOXO3a*) to a role in increased invasiveness (*ERBB3*).

The similarity of our findings with treatment of the panel of cell lines with trastuzumab, and the combination treatment of lapatinib with capecitabine, further strengthen the hypothesis that this gene expression profile is indicative of the HER2 pathway being inhibited. Both treatments provided similar expression patterns at 12 hours post treatment. It should also be noted that despite the addition of the chemotherapeutic agent capecitabine to the lapatinib treatment, the gene expression profile remained evident. To examine if the gene changes are stable over a longer period of time, the cell lines were treated for 36 hours with the 1 μM lapatinib, 150 nM afatinib and 150 nM neratinib. The differential expression of the genes were examined and compared to the differential expression exhibited at 12 hours. The trends that were exhibited 12 hour post treatments were also seen 36 hour post treatments. These results provide a strong indicator that expression changes in this panel of genes is a good and robust representation of responsiveness not only to lapatinib but also afatinib and neratinib.

To evaluate if this gene panel is only responsive to HER2-targeted therapies, the panel of cell lines (BT474, SKBR3 and MDAMB453) were also treated with 1 μM gefitinib. Gefitinib is a EGFR inhibitor that is used in the treatment non-small cell lung cancer [23]. The panel of cell lines examined have a variable level of EGFR expression. MDAMB453 do not express any EGFR [24] with BT474 expressing low levels [25] and SKBR3 expressing intermediate levels [24]. BT474 and SKBR3 are both sensitive to gefitinib [26]. The trend that was observed in response to gefitinib did not correlate with that shown in response to the HER2-targeting TKIs, giving a strong indication that this gene expression trend is associated with response to HER2 and not EGFR inhibition. Cells were also treated with 1 μM dasatinib, a BCR/ABL and src inhibitor and 25 nM epirubicin for 12 hours. Acting as control treatments, the observation that there was no similarities in the gene expression profile exhibited following these treatments, allows us to assume that it is the inhibition of the HER2 pathway that gives rise to this profile and not the induction of apoptosis using unspecific targeted or chemotherapeutic agents.

Although all of the genes in this panel have been reported to have roles in breast cancer [17], there have been no reports of expression changes in *NR3C1* and *RB1CC1* genes in response to afatinib, neratinib or gefitinib. *FOXO3A* expression changes have not been reported to change in response to neratinib or afatinib. However, there are a small number of publications that have indicated that gefitinib can target *FOXO3A* and thereby mediate cell cycle arrest and apoptosis in breast cancer [27-29]. *ERBB3* has not been studied in combination with neratinib treatment and very limited information regarding the effects of afatinib on the expression of this gene is available [11]. With regards to gene expression changes in response to gefitinib treatment, some results have been published that contradict the results of this study [30]. Groval *et al*., have indicated that treatment with 5 μM gefitinib for 48 hours resulted in an increase in the expression of *ERBB3* gene in both SKBR3 and BT474 cell lines. *CCND1* expression changes have not been reported for cellular responses to neratinib or afatinib, however, there is some data in the literature demonstrating that treatment with gefitinib can result in down-regulation of *CCND1* which supports our finding [31,32].

## Conclusions

In this study, we further investigated the previous characterisation of a five gene expression response that had emerged with a single 12 hour treatment of sensitive cells with a pharmacologically relevant concentration of lapatinib. This profile was a powerful predictor of the overall post treatment sensitivity of the cell lines to this agent. The addition of the active metabolite of capecitabine to lapatinib treatment did not mask the predictive value or specificity of the gene expression profile; suggesting that such a clinically relevant drug combination would not impede the characteristic lapatinib exposure gene expression response.

This profile was also evaluated in response to further HER2 targeting therapies; afatinib, neratinib and traztuzumab and over longer durations of up to 36 hrs. The gene expression changes in response to gefitinib were examined to determine if the response was associated with the HER2 inhibition or potentially a HER2/EGFR inhibition. Dasatinib and epirubicin treatments were also used to evaluate the specificity of the panel of genes and there correlation with the inhibition of the HER2 pathway. When compared with the gene expression profile previous described, [17] the afatinib, neratinib and traztuzumab profiles exhibited similar trends in the differential expression of *RB1CC1*, *FOXO3A*, *NR3C1*, *ERBB3* and *CCND1* following a 12 hour exposure with 150 nM in the three breast cancer cell lines tested; BT474, SKBR3 and MDAMB453, while no such correlation was evident with treatment by the EGFR-targeting agent, gefitinib or that with BCR/ABL and Src inhibitor, dasatinib, or the anthracycline cytotoxic agent, epirubicin.

Taken together, our findings indicate that the identified gene expression profile is characteristic of the sensitivity of the cells to HER2-inhibitor treatment, robust over time, is consistent when examined in the clinically relevant combination of lapatinib with capecitabine and is also more broadly characteristic of a HER2-inhibitory response, than simply a lapatinib response alone. The gene expression changes also clearly identify and predict treatment response to afatinib, neratinib and trastuzumab in breast cell lines. This suggests that examination of the changes in the expression of these five genes after exposure to HER2-targetting therapies could have significant predictive value for overall tumour response. Following on from this *in vitro* investigation, there will be both cell line xenograft and HER2+ patient derived xenograft (PDX) studies carried out to examine and validate the gene expression profile changes in the *in vivo* environment. If such a pattern change is evident in responsive human tumours it would have huge potential to rapidly identify patients getting clinical benefit for such treatments.

## Competing interests

The authors declare that they have no competing interests.

## Authors’ contributions

SFM performed all of the bioinformatic/statistical analysis. FON treated the cells with targeted therapies extracted the RNA and performed Taqman RT PCR and the proliferation assay. STA participated in RNA extraction and TaqMan RT PCR and analysis and interpretation of the results. FON, SFM, STA, JC, MC ROC and PD contributed to the result interpretation and manuscript preparation. ROC and STA equally conceived the study, participated in its design, coordination and interpretation of the results and finalized the manuscript. All authors read and approved the final manuscript.
